# Histone H1 interphase phosphorylation becomes largely established in G_1 _or early S phase and differs in G_1 _between T-lymphoblastoid cells and normal T cells

**DOI:** 10.1186/1756-8935-4-15

**Published:** 2011-08-05

**Authors:** Anna Gréen, Bettina Sarg, Henrik Gréen, Anita Lönn, Herbert H Lindner, Ingemar Rundquist

**Affiliations:** 1 Division of Cell Biology, Department of Clinical and Experimental Medicine, Linköping University, SE-58185 Linköping, Sweden; 2 Clinical Pharmacology, Division of Drug Research, Department of Medical and Health Sciences, Linköping University, SE-58185 Linköping, Sweden; 3 Division of Clinical Biochemistry, Biocenter, Innsbruck Medical University, Austria

## Abstract

**Background:**

Histone H1 is an important constituent of chromatin, and is involved in regulation of its structure. During the cell cycle, chromatin becomes locally decondensed in S phase, highly condensed during metaphase, and again decondensed before re-entry into G_1_. This has been connected to increasing phosphorylation of H1 histones through the cell cycle. However, many of these experiments have been performed using cell-synchronization techniques and cell cycle-arresting drugs. In this study, we investigated the H1 subtype composition and phosphorylation pattern in the cell cycle of normal human activated T cells and Jurkat T-lymphoblastoid cells by capillary electrophoresis after sorting of exponentially growing cells into G_1_, S and G_2_/M populations.

**Results:**

We found that the relative amount of H1.5 protein increased significantly after T-cell activation. Serine phosphorylation of H1 subtypes occurred to a large extent in late G_1 _or early S phase in both activated T cells and Jurkat cells. Furthermore, our data confirm that the H1 molecules newly synthesized during S phase achieve a similar phosphorylation pattern to the previous ones. Jurkat cells had more extended H1.5 phosphorylation in G_1 _compared with T cells, a difference that can be explained by faster cell growth and/or the presence of enhanced H1 kinase activity in G_1 _in Jurkat cells.

**Conclusion:**

Our data are consistent with a model in which a major part of interphase H1 phosphorylation takes place in G_1 _or early S phase. This implies that H1 serine phosphorylation may be coupled to changes in chromatin structure necessary for DNA replication. In addition, the increased H1 phosphorylation of malignant cells in G_1 _may be affecting the G_1_/S transition control and enabling facilitated S-phase entry as a result of relaxed chromatin condensation. Furthermore, increased H1.5 expression may be coupled to the proliferative capacity of growth-stimulated T cells.

## Background

Cell division is a complex process, in which correct passage through the cell cycle is essential for cell survival and correct transmission of genetic information to the daughter cells. During the cell cycle, the cell nucleus undergoes dramatic structural changes. DNA, which is compacted into chromatin by various proteins, is locally decondensed in S phase, but condenses in prophase. In metaphase, highly condensed chromosomes are visible, which start to segregate during anaphase. Segregation is completed during telophase, and two daughter cells are produced. Before re-entry into G_1_, the chromatin again becomes dispersed.

In the nucleosome, the basic unit of chromatin, approximately 146 bp of DNA are wrapped 1.65 turns around an octamer consisting of two copies of each core histone: H2A, H2B, H3 and H4 [1]. A fifth histone, histone H1 (also referred to as linker histone), binds at or near to the entry/exit point of DNA and to linker DNA [2]. Histone H1 has a central globular domain and hydrophilic tails in the N and C terminals. Histone H1 is a protein family with at least eight members in mammals. Some of these are present only in highly specialized cell types. In most somatic cells, histones H1.2, H1.3, H1.4 and H1.5 are present [3]. The function of histone H1 in the cell and the purpose of several H1 subtypes remain to be determined in detail; however, histone H1 is implicated in the compaction of chromatin into higher-order structures [4] and in transcriptional regulation [3,5-7]. Knockout experiments in mice have identified a remarkable redundancy and overlapping functionalities of the different subtypes, but have also proved that histone H1 is indispensable in mouse development [8]. In addition, some subtypes seem to have specialized functions [9]; a particular example is H1.2, which is a part of the apoptosis signaling process as a response to DNA double-strand breaks [10].

In addition to the complexity of multiple subtypes, H1 subtypes are post-translationally modified, primarily by phosphorylation at multiple sites. The significance of this modification is unclear, but is believed to reduce the affinity of histone H1 for chromatin [11,12]. Histone H1 phosphorylation has been implicated in various physiological processes, for example in gene regulation, chromatin condensation/decondensation, and cell-cycle progression [12]. Regulation of gene expression may be executed through chromatin remodeling, regulated by histone H1 phosphorylation [13,14].

H1 phosphorylation was initially connected to mitotic condensation of chromatin [15], but other studies have shown that H1 phosphorylation can also be involved in decondensation of chromatin [11]. Increasing evidence suggests that histone H1 phosphorylation is involved in both chromatin condensation and decondensation during the cell cycle. In mid to late G_1 _and S phase, increased H1 phosphorylation, Cdk2 activation and local chromatin decondensation occur [16,17]. This may be performed by disassembly of heterochromatin, as H1 phosphorylation by Cdk2 disrupts the interaction between histone H1 and heterochromatin protein 1α [18]. The phosphorylation of histone H1 and chromatin decondensation in mid to late G_1 _and S phases have been suggested to be a prerequisite for DNA-replication competence [12,16,19,20].

The phosphorylation of H1 histones in the cell cycle has been described as a sequential event. In Chinese hamster cells, and in rat and mice synchronized cell cultures, H1 phosphorylation was shown to start during mid to late G_1_, increase during S, and reach its maximum at mitosis [21,22]. The major phosphorylation sites in human somatic H1 histones have been mapped and are located on serines in SP(K/A)K motifs in H1.2, H1.3, H1.4 and H1.5 during interphase [23]. Mitotic up-phosphorylation takes place on threonine residues only [23,24].

Increased H1 phosphorylation in *ras*-transformed G_1 _mouse fibroblasts, compared with their normal counterparts, has been detected [25]. The increase in detected mouse H1b (homologous to human H1.5) phosphorylation in the transformed cells was concluded to be caused by increased Cdk2 activity in the transformed mouse fibroblasts [26]. Furthermore, in Rb-deficient fibroblasts, increased H1 phosphorylation was detected in G_1 _along with less condensed chromatin and increased Cdk2 activity [17].

In the search for cell cycle-specific phosphorylation of histone H1, human cancer cells or cells from species other than human have been used. To our knowledge, no normal human cells have been investigated to date. Because many signaling pathways are dysregulated in cancer cells, especially within the cell-cycle control system, it is of interest to use normal cells when studying cell-cycle events. In addition, most other studies have used various chemical agents to arrest or synchronize the cycling cells in the different cell-cycle stages. Because such methods may affect H1 phosphorylation, we used activated T cells and fluorescence-activated cell sorting for studying the cell cycle-dependent phosphorylation of human H1 histones. To detect differences in the phosphorylation pattern between malignant and normal cells, the cell cycle-dependent H1 phosphorylation of Jurkat T lymphoblastoid cells was also examined. Histone H1 subtype composition and phosphorylation was analyzed by reversed phase high performance liquid chromatography (RP-HPLC) and capillary electrophoresis. We found a substantial increase in H1.5 content after activation of T cells. Furthermore, the major part of interphase H1 phosphorylation took place in G1 or early S phase, and was preserved during S and G2/M phases. We also found enhanced H1 phosphorylation, in particular for H1.5, in the G_1 _phase of T-lymphoblastoid cells compared with activated normal T cells.

## Results

### T-cell activation results in rapidly proliferating T-cell populations

**Figure 1:**
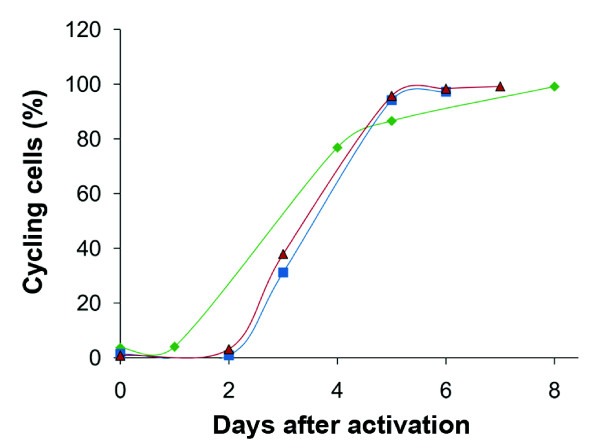
**T-cell activation assessed by 5(6)-carboxyfluorescein diacetate N-succinimidyl ester (CFSE) tracing**. The proportion of cycling cells, with decreased CFSE fluorescence, was measured on days 0, 1, 4, 5 and 8 for sample 1 (green); on days 0, 2, 3, 5 and 6 for sample 2 (blue); and on days 0, 2, 3, 5, 6 and 7 for sample 3 (red). Sorting of activated T cells was performed on day 8, 6 and 7, respectively.

After isolation, the peripheral blood lymphocytes (PBLs) from all three donors consisted of over 94% viable cells, as measured by Annexin V. They appeared to contain a normal T-cell ratio, which was confirmed by measuring the fraction of CD3+ cells. Cell division started after 2 days of activation, and was evident at day 3 (Figure 1). Upon sorting, more than 97% of the cells were passing through the cell cycle (Figure 1).

At this point most cells had very low levels of 5(6)-carboxyfluorescein diacetate N-succinimidyl ester (CFSE) fluorescence, as a result of multiple cell divisions. In addition, cell-cycle analysis using propidium iodide (PI) staining showed appearance of S and G_2 _cells at day 2 and thereafter. During cultivation of PBLs, the fraction of CD3-positive cells increased, and at the time for cell sorting, all cell populations consisted almost solely of T cells (data not shown). When stimulated, CD4+ and CD8+ T cells proliferate, whereas other cell types die through apoptosis or become diluted via recultivation of the growing T cells. On examination of the activated PBLs under the microscope, samples from all donors were found to have a similar cell appearance (data not shown). At sorting, the T-cell cultures consisted of more than 90% viable cells. The cell-cycle distributions of activated T cells are presented in Table 1.

**Table 1:** Cell-cycle phase distributions of activated T-cell populations, determined by flow cytometry using PI staining, at the time of cell sorting

Sample	G_1_, %	S, %	G_2_/M, %
T cells 1	63.8	29.6	6.6
T cells 2	72.6	23.1	4.3
T cells 3	72.8	23.4	3.8

### H1.5 expression is increased in proliferating T cells compared with resting lymphocytes

**Figure 2:**
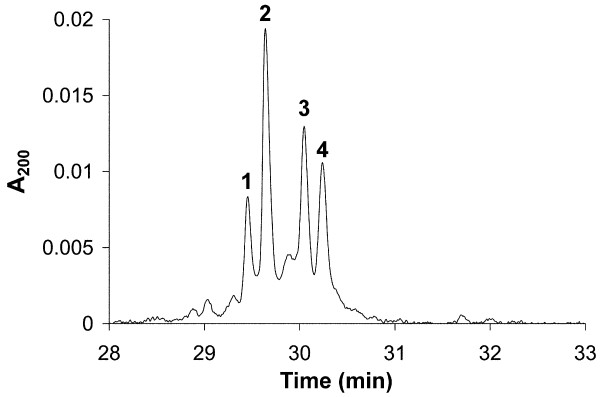
**High-performance capillary electrophoresis (HPCE) separation of perchloric acid extracted H1 histones from non-activated peripheral blood lymphocytes (PBLs)**. Only unphosphorylated subtypes were detected. The peak designations are (1) H1.5, (2) H1.4, (3) H1.3 and (4) H1.2.

The H1 subtype composition in non-activated, resting (G_0_) PBLs was analyzed by high-performance capillary electrophoresis (HPCE) (Figure 2). The migration order coincided exactly with previously published data [27], and no other peaks were detected. The relative subtype compositions were then determined by measuring the height of the peaks containing H1.2, H1.3, H1.4 and H1.5 in the electropherograms, and normalizing these to the sum of these peak heights. The relative H1 subtype composition in PBLs from the three donors was (mean ± SD): 18.8 ± 2.1% for H1.2, 25.9 ± 2.7% for H1.3, 39.7 ± 3.9% for H1.4, and, 15.6 ± 0.7% for H1.5. This subtype composition is presumed to be approximately the same as in pure T-cell populations, because PBLs from normal donors generally contain more than 80% T cells, with the major part of the contaminating cells known to be B cells. We have previously investigated the H1 subtype distribution in purified human B cells, and these results showed an almost identical H1 subtype composition to that of the PBLs described above (unpublished data).

**Figure 3:**
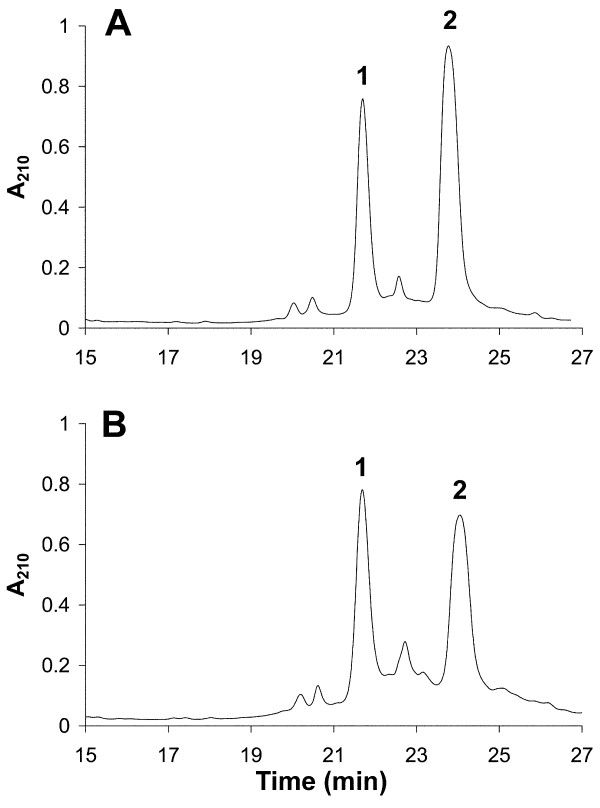
**Reversed phase high performance liquid chromatography (RP-HPLC) fractionation of H1 histones from (A) activated T cells and (B) Jurkat cells**. Peak 1 contained H1.5 and phosphorylated variants thereof, and peak 2 contained subtypes H1.2, H1.3, H1.4 and their phosphorylated counterparts.

At the time for cell sorting, a significant relative increase in H1.5 content was seen in activated T cells from all donors, compared with G_0 _cells. This is illustrated by RP-HPLC separation of H1 proteins extracted from activated T cells from donor 1, shown in Figure 3A, while the corresponding RP-HPLC fractionation of H1 from Jurkat cells is presented in Figure 3B. The areas of the peaks containing H1.5 and the peaks containing the remaining subtypes were determined for both activated T cells and Jurkat cells. The small peak between peaks 1 and 2, most probably containing H1x, was omitted from the calculations. The relative H1.5 content was determined to be 36 ± 2% (n = 3) for activated T cells, and 47 ± 1% (n = 3) for Jurkat cells. The available number of resting T cells from each donor was not sufficiently large for growth stimulation and RP-HPLC fractionation, but because both RP-HPLC and HPCE use UV absorption for protein detection, and we only report the fractions of each subtype or group of subtypes, these results can be compared.

### Proliferating T cells and Jurkat cells contain multiple phosphorylated H1 subtypes

**Figure 4:**
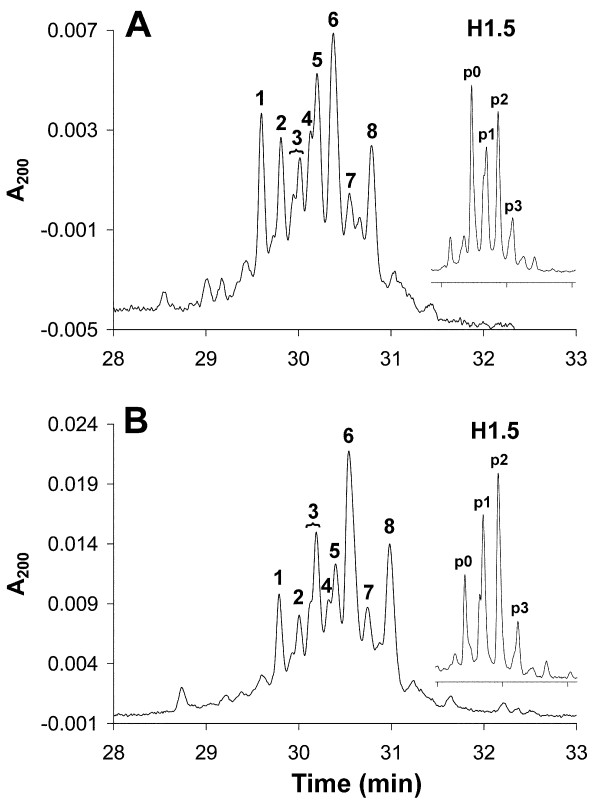
**High-performance capillary electrophoresis (HPCE) separations of H1 histones and reversed phase high performance liquid chromatography (RP-HPLC)-fractionated H1**.5** (inset) from (A) activated T cells, and (B) exponentially growing Jurkat cells.** The peaks were identified as: (1), unphosphorylated H1.5; (2) unphosphorylated H1.4; (3) monophosphorylated H1.5; (4) monophosphorylated H1.4; (5) unphosphorylated H1.3; (6) diphosphorylated H1.5, together with unphosphorylated H1.2 and possibly diphosphorylated H1.4; (7) monophosphorylated H1.3; and (8) monophosphorylated H1.2 together with triphosphorylated H1.5.

H1 samples were extracted from cycling, activated T cells. HPCE separation of H1 histones displayed the presence of multiple peaks due to phosphorylation in addition to the unphosphorylated subtypes (Figure 4A). Exponentially growing Jurkat cells displayed a somewhat increased level of H1 phosphorylation (Figure 4B), compared with any T-cell sample. All migration orders coincided exactly with previously published data [27]. The differences between T cells and Jurkat cells were also shown by the H1.5 phosphorylation patterns obtained after RP-HPLC separation prior to HPCE (Figure 4, insets).

### Flow sorting of T cells and Jurkat cells in different cell-cycle phases

**Figure 5:**
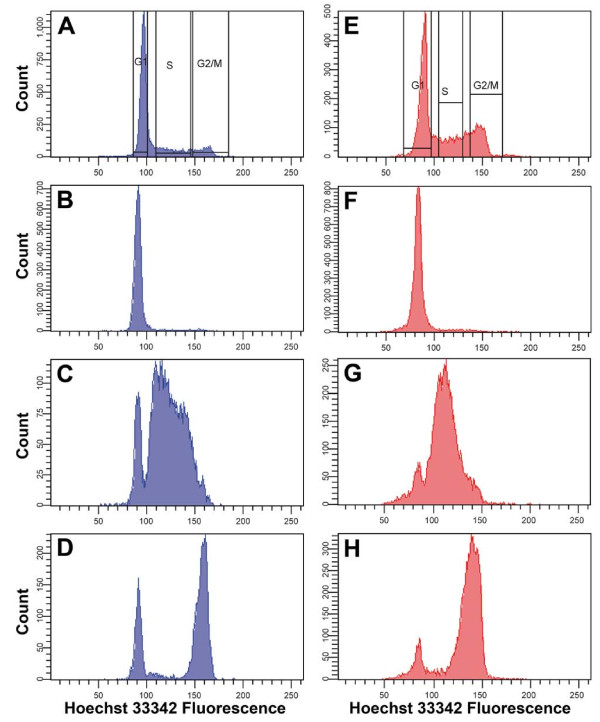
**DNA histograms with sorting gates of Hoechst 33342-stained (A) T cells and (E) Jurkat cells**. After cell sorting, the different cell populations were reanalyzed. **(B-D) **Reanalysis of sorted T-cell populations in **(B) **G_1_, **(C) **S, and **(D) **G_2_/M populations. **(F-H) **Reanalysis of sorted Jurkat populations in **(F) **G_1_, **(G) **S, and **(H) **G_2_/M populations.

Flow-sorting DNA histograms (with sorting gates for G_1_, S and G_2_/M populations) of cycling T cells and Jurkat cells are shown in Figure 5. The sorted populations were reanalyzed after sorting to check the purity of the different populations (Figure 5). Flow sorting of Jurkat cells resulted in almost pure cell-cycle populations (Table 2). Sorting of cycling T cells resulted in relatively pure G_1 _and S populations, but there was some cross-contamination of the G_2_/M populations seen during reanalysis, primarily by cells with a measured DNA content corresponding to G_1 _cells (Table 2 and Figure 5). In addition, one of the T-cell samples (T cells 3) had a higher G_1 _cross-contamination of the S-phase cells (Table 2) than did the other T-cell samples. This can be explained by an increase in the spreading of flow-sorting droplets in this particular experiment.

**Table 2:** Purity of flow-sorted populations from activated T cells and Jurkat cells

	Purity, %		
	
Sample	G_1_	S	G_2_/M
T cells 1	96.6	88.4	63.6
T cells 2	95.9	83.4	70.5
T cells 3	92.1	71.0	60.9
Jurkat 1	94.8	88.0	89.3
Jurkat 2	93.1	86.1	87.7
Jurkat 3	93.4	88.5	86.2

**Figure 6:**
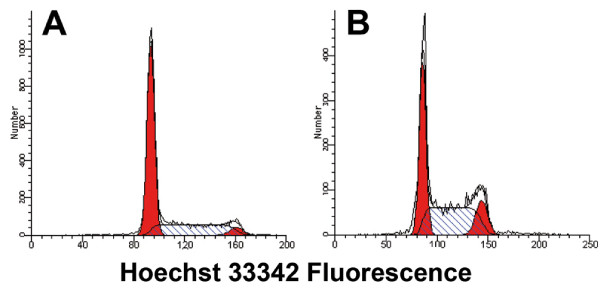
**Cell-cycle analysis of DNA histograms after gating in forward scatter (FSC) and side scatter (SSC) and doublet discrimination**. (A) Activated T cells, (B) Jurkat cells.

The cell-cycle distribution of the DNA histograms from Hoechst 33342-stained cells at flow sorting was determined using Modfit (Figure 6). Cell-cycle data are presented in Table 3. From these data, it is evident that there were fewer T cells in G_2_/M compared with Jurkat cells. This may be an explanation for the lower purity of the sorted G_2_/M populations from T cells.

**Table 3:** Cell-cycle distribution of cell populations stained with Hoechst 33342 selected for sorting (after gating in forward/side scatter (FSC/SSC) and doublet discrimination)

Sample	G_1_, %	S, %	G_2_/M, %
T cells 1	58.9	35.3	5.8
T cells 2	64.7	31.0	4.3
T cells 3	69.6	26.4	4.0
Jurkat 1	51.4	33.1	15.5
Jurkat 2	41.2	44.7	14.1
Jurkat 3	50.3	38.2	11.5

### The phosphorylation of H1 histones starts in the G_1 _phase of the cell cycle in normal proliferating T cells

**Figure 7:**
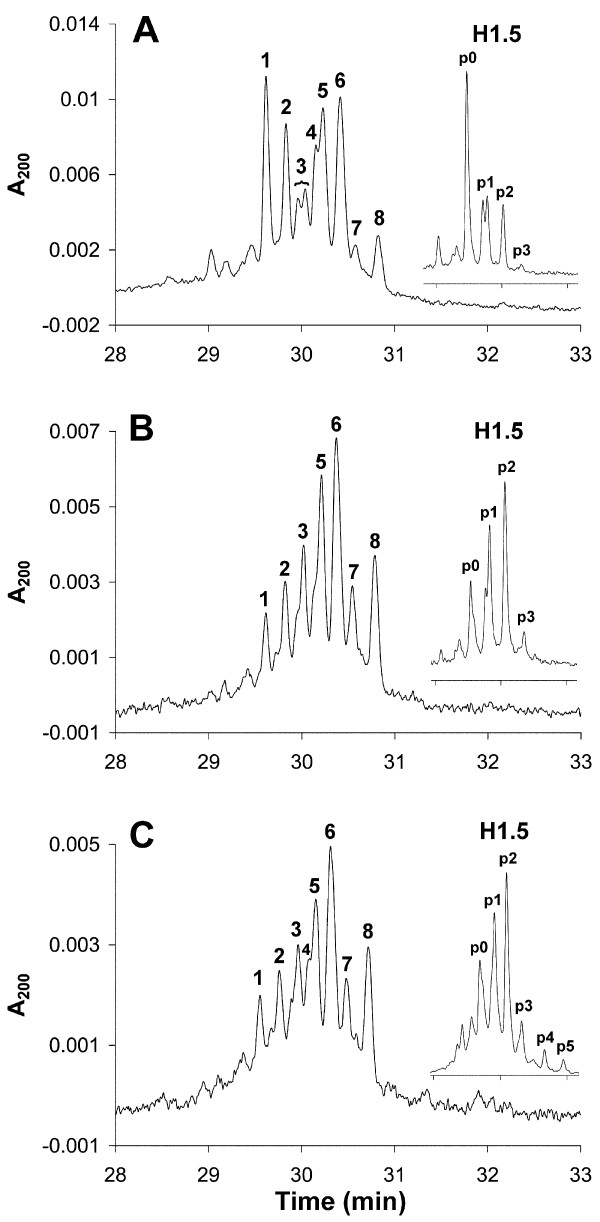
**High-performance capillary electrophoresis (HPCE) separations of H1 histones and reversed phase high performance liquid chromatography (RP-HPLC)-fractionated H1**.**5 (insets) extracted from activated, flow-sorted T cells.** Cells in **(A) **G_1_, **(B) **S, **(C) **G_2_/M phase. Peak designations as in Figure 4.

The Histone H1 subtype and phosphorylation pattern was determined using HPCE for G_1_, S and G_2_/M T-cell populations (Figure 7). Only small variations were detected between the three T-cell samples. Furthermore, H1.5 phosphorylation was also examined after RP-HPLC separation followed by HPCE of the isolated H1.5 peak from the RP-HPLC fractionation of H1 histones (insets in Figure 7).

In G_1 _T cells, approximately 50% of H1.5 was present in its unphosphorylated form (as determined by peak heights in the inset in Figure 7A). Most of the remaining H1.5 was either mono- or diphosphorylated. The same pattern is probably to be true also for H1.4, but this cannot be verified due to the co-migration of diphosphorylated H1.4 with unphosphorylated H1.2 and diphosphorylated H1.5 (peak 6 in Figure 7). H1.2 monophosphorylation was evident (peak 8 in Figure 7A). The level of H1.3 phosphorylation was low (peak 7 in Figure 7A).

Cells in S phase had more extended H1.5 phosphorylation, with a clear increase in mono-, di- and triphosphorylated H1.5 (inset in Figure 7B). A clear reduction of unphosphorylated H1.5 was evident (peak 1). Histone H1.4 phosphorylation also increased, which was seen through reduction of the peak containing unphosphorylated H1.4 (peak 2 in Figure 7B). H1.2 and H1.3 monophosphorylation increased.

The S-phase phosphorylation pattern was largely preserved in the sorted G_2_/M T-cell populations (Figure 7C). It was evident that the extent of H1.5 mono- and diphosphorylation was preserved, whereas a small increase in triphosphorylated H1.5 could be detected. In addition, the presence of p4 and p5 hyperphoshorylated forms was indicated during G_2_/M. These phosphorylations probably originate from the metaphase cells in this population, because these forms have been detected previously in mitotic CEM cells [23]. However, we could not detect higher phosphorylation forms of the other subtypes, although they are predicted to be present in metaphase cells. This finding, and that of the low amounts of tetra- and pentaphosphorylated forms of H1.5, can probably be explained by the relatively short time during mitosis when these forms occur. Further studies are needed to address the issue of mitotic phosphorylation.

### Exponentially growing Jurkat cells contain more extensively phosphorylated H1 subtypes in the G_1 _phase of the cell cycle compared with activated T cells

After flow sorting of exponentially growing Jurkat cells, H1 histones from G_1_, S and G_2_/M cell populations were extracted and separated by HPCE. The H1 subtype and phosphorylation pattern was reproducible between the Jurkat samples.

**Figure 8:**
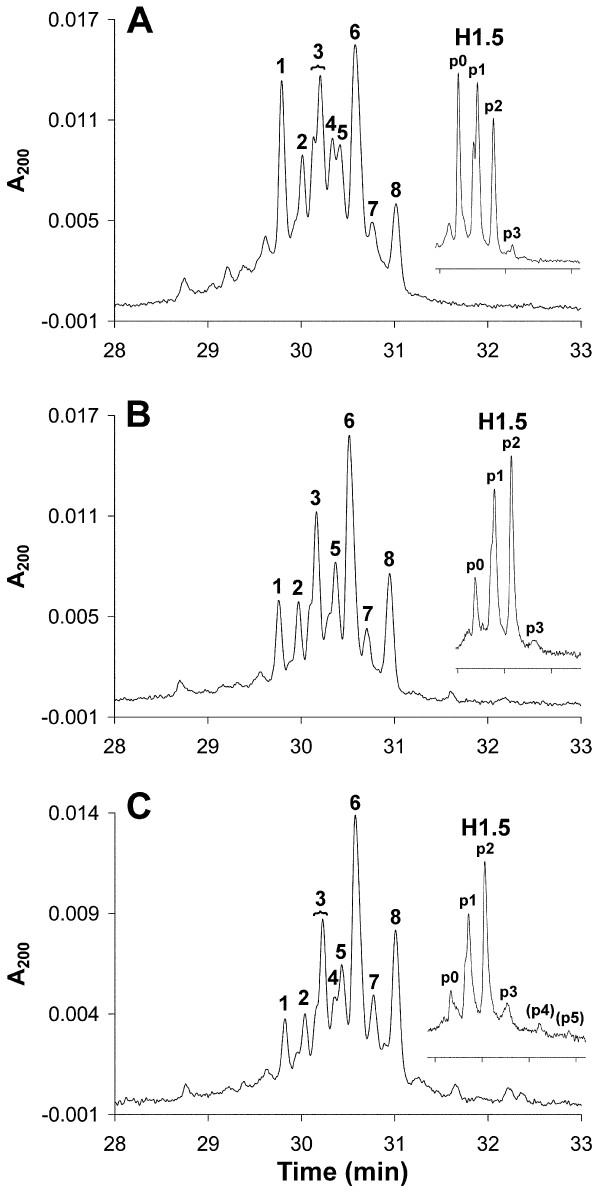
**High-performance capillary electrophoresis (HPCE) separations of H1 histones and reversed phase high performance liquid chromatography (RP-HPLC)-fractionated H1**.**5 (insets) from flow-sorted Jurkat cells.** Cells in **(A) **G_1_, **(B) **S, **(C) **G_2_/M phase Peak designations as in Figure 4.

In G_1 _Jurkat cells, highly phosphorylated H1.5 was detected (Figure 8A, inset). Histone H1.4 monophosphorylation was evident (Figure 8A, peak 4), and possibly diphosphorylated H1.4 was present as a part of peak 6. H1.2 monophosphorylation was detected (Figure 8, peak 8). The level of H1.3 phosphorylation was low (Figure 8, peak 7).

In Jurkat cells sorted from S phase, H1.5 phosphorylation increased substantially. The level of unphosphorylated H1.4 (Figure 8B, peak 2) decreased slightly, whereas monophosphorylated H1.4 (Figure 8A, peak 4; appearing only as a shoulder in Figure 8B, peak 5) decreased, probably due to an increase in diphosphorylated H1.4. H1.2 monophosphorylation was increased (Figure 8B, peak 8), whereas H1.3 phosphorylation was virtually unaffected (Figure 8B, peak 7).

In G_2_/M, the H1 phosphorylation pattern resembled that in S phase, but the extent of phosphorylation increased somewhat for all subtypes (Figure 8C). This is also evident from Figure 8C (inset), in which unphosphorylated H1.5 decreased and higher phosphorylated forms were detected (p4 and p5). The purity of the sorted G_2_/M cells (Table 2) was high, but some late S-phase cells might still have been present in these samples (Figure 6B).

The major difference between activated T cells and Jurkat cells was a more extended phosphorylation in G_1 _Jurkat cells. In addition, G_2_/M Jurkat cells (Figure 8C) contained a lower level of unphosphorylated H1.5 compared with G_2_/M T cells (Figure 7C). However, this difference may be explained by a contamination of G_1 _cells in the sorted G_2_/M T-cell populations (Figure 5D), resulting in an underestimation of G_2_/M phosphorylation. Therefore, we anticipate that T cells and Jurkat cells exhibit an almost similar H1 phosphorylation pattern in S phase (Figure 7B; Figure 8B) and in G_2_/M phase (Figure 7C; Figure 8C).

## Discussion

Cell-cycle regulation is important in normal tissue homeostasis and both in the origin and progression of cancer. A vital part of cell-cycle regulation and progression is the preparation of chromatin for replication. We and others believe that H1 histones and their phosphorylation are important in these processes. In this study, we found that the interphase phosphorylation pattern of H1 histones was established in G_1 _or early S phase in activated human T cells and Jurkat cells. This pattern was largely preserved during S and G_2_/M phases. Unfortunately, because of a lack of cells, we were not able to introduce separate sorting windows in early and late S phase, but because H1 phosphorylation has been shown to occur site-specifically in a certain order [23], it is unlikely that rapid dephosphorylation/rephosphorylation events affecting different phosphorylation sites can be an alternative explanation for the preserved phosphorylation patterns. Activation of T cells altered the H1 subtype composition; in particular, we detected a significant increase in the relative H1.5 content in cycling T cells compared with resting T cells.

The pattern of H1.5 mono- and diphosphorylation and of H1.2 and H1.3 monophosphorylation (and most probably of H1.4 mono- and diphosphorylation) became to a large extent established in G_1 _phase or early S phase, and remained virtually preserved in G_2_/M in both activated T cells and Jurkat cells. The similarity between S-phase and G_2_/M-phase phosphorylation patterns also indicate that the newly synthesized H1 histones in S phase became phosphorylated to the same extent as the pre-existing ones, in line with previous data. The small differences in G_2_/M phosphorylation patterns between T cells and Jurkat cells can be explained by the higher content of contaminating G_1 _cells in the T-cell G_2_/M populations. The G_1 _phosphorylation pattern differed between Jurkat and activated T cells, with more extended phosphorylation in G_1 _Jurkat cells. We expect that all these phosphorylations occur on serine residues, because it has previously been shown that only serines in SP(K/A)K motifs were phosphorylated in interphase [23,24]. The number of S/TPXK sites, and their phosphorylation, in the present H1 subtypes has been thoroughly investigated previously, and our results did not deviate from those results [23]. No influence on other sites was detected.

Our observations are partly in contrast with earlier data describing a sequential increase of H1 phosphorylation across the cell cycle [21,22,28]. In mouse NIH 3T3 fibroblasts, H1 phosphorylation began during late G_1_, increased during the S phase, and in late S phase 0 to 3 phosphate groups were detected on various mouse H1 subtypes [22]. In the G_2_/M transition, H1 phosphorylation levels increased, and reached their maximum at M phase [22]. Using Chinese hamster cells, with one predominant histone H1 subtype, histone H1 was shown to have no phosphate groups in early G_1 _[28]. Phosphorylation began in mid G_1 _[21], and one phosphate group was detected in the beginning of S phase [28]. During the S and G_2 _phases, up to three phosphates were seen, and maximum was reached at M phase, with up to six phosphates [21,28].

In agreement with previous data, our results indicate that in normal T cells, H1.2, H1.3, H1.4 and H1.5 are mainly unphosphorylated at the beginning of the G_1 _phase of the cell cycle. This is probably true also after T-cell activation, as H1 histones from slow-growing populations of T cells contained very few phosphorylated variants (data not shown). However, some caution should be taken in data interpretation from such T-cell cultures because these cells may be on the way to become apoptotic, even though only viable cells were sorted. We have recently shown that apoptosis may affect the H1 phosphorylation pattern [27].

**Figure 9:**
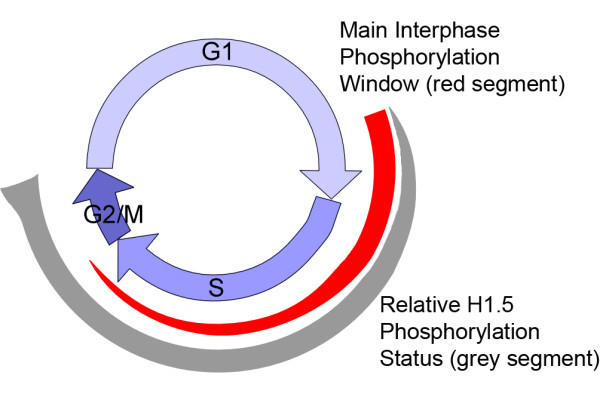
**Hypothetical model for cell cycle-dependent histone H1.5 serine phosphorylation in T cells and Jurkat cells**. The main kinase activity takes place during late G_1 _and early S phase as indicated by the red segment. The relative phosphorylation level, shown as the fraction of phosphorylated H1.5 serines per DNA unit, is indicated by the width of the grey segment. For clarity, the threonine phosphorylation taking place during mitosis is shown as a widening of the grey segment at the end of the cell cycle. The obtained results on H1.5 phosphorylation fit with, but do not prove, this model.

H1 histones are conserved proteins, and require strongly resolving analytical techniques for their separation [29]. In addition, the presence of differentially phosphorylated subtypes further complicates the separation of all variants. However, the combination of RP-HPLC and HPCE allows complete resolution of H1.5 and its phosphorylated forms. From our data, we thus propose the following model of cell cycle-dependent serine phosphorylation of histone H1.5 (Figure 9), but it may also be valid for other H1 subtypes.

This model indicates that H1.5 is unphosphorylated during the first part of the G_1 _phase, and becomes mono- and diphosphorylated on serine residues later in G_1 _and in early S phase. It is possible that some H1.5 monophosphorylated at Ser17 is present already in earlier stages of the G_1 _phase, as indicated by recent data [24]. Besides the complementary phosphorylation of the newly synthesized H1 molecules during S phase (Figure 9), some further up-phosphorylation takes place during S, G_2 _and M phases. In particular, threonine phosphorylation during mitosis results in a slight widening of the relative amount of phosphorylated H1.5 sites at the end of the cell cycle (Figure 9, grey segment) before the expected complete dephosphorylation takes place before the next G_1 _phase is entered.

The differences we detected in G_1 _phosphorylation between T cells and Jurkat cells may be explained either by the shorter cell-cycling time in Jurkat cells, and/or by increased kinase activity. Rapid cell growth usually correlates with a shorter G_1 _phase, and thereby more G_1 _cells within the phosphorylation window for Jurkat cells compared with T cells, and a higher degree of phosphorylated H1.5. The fraction of cells in S phase is often used as a measurement of cell-cycle velocity. In activated T cells from the three donors, the average fraction of S phase cells was 31%, compared with 39% in the three Jurkat cell samples. Therefore, the difference in cell-cycling time is probably not sufficiently large to be the sole explanation for the differences in G_1 _phosphorylation.

Another explanation is the presence of an overactive H1 kinase in the G_1 _phase of Jurkat cells. H1 from *ras*-transformed mouse fibroblasts exhibited higher phosphorylation than did their untransformed counterparts [25]. This was not a result of cell-cycle changes upon transformation, because transformed G_1_/S arrested cells had higher levels of phosphorylated H1 than G_1_/S arrested untransformed cells [25]. The *ras*-transformed cells also had less condensed chromatin than in untransformed cells [25]. In further studies, the increased H1b (homologue to human H1.5) phosphorylation after *ras *transformation of mouse fibroblasts was found to be derived from overactivity of Cdk2, rather than from reduced activity of H1 phosphatases [26]. In the *ras*-transformed mouse fibroblasts, *ras *expression resulted in an initial increase in p21^cip1 ^(a Cdk2 inhibitor) levels and inhibition of Cdk2 activity, followed by a decrease in p21^cip1 ^and activation of Cdk2, producing increased H1b phosphorylation [26]. Transformation of mouse fibroblasts with other oncogenes affecting the Ras-mitogen-activated protein kinase signal-transduction pathway (for example *mos, raf, fes *and *myc*) also resulted in increased H1 phosphorylation [25]. Therefore, we suggest that a part of the increased H1.5 phosphorylation in G_1 _in Jurkat cells is a result of overactive H1 kinases, either within an unchanged phosphorylation window, or during an extended phosphorylation window occupying a larger part of the G_1 _phase. An alternative explanation for the extended G_1 _H1.5 phosphorylation would be a defective H1 phosphatase. In agreement with previous data [26], this is less likely, because the sorted G_1 _cells contained substantially reduced levels of phosphorylated H1 compared with G_2_/M populations.

In G_1 _and S phases, H1 phosphorylation is coupled to less condensed chromatin [12,17,25]. Extended H1 phosphorylation may then lead to facilitated S-phase entry of malignant cells, as part of a disturbed cell-cycle control. Increasing evidence indicates that histone H1 phosphorylation in S phase is important for chromatin decondensation in the replication process [16]. Possibly, the specific serine phosphorylation pattern established in late G_1_/early S phase, as described here, takes place to partially displace certain parts of the H1 histones to allow access for, or to recruit, other proteins that are involved in chromatin decondensation and S-phase progression, as described for cdc45 [16]. The fine-tuning of replication timing during S phase may then be regulated by small additional local variations in the H1 phosphorylation pattern, in line with recent observations [20].

The precise physiological role of histone H1, its phosphorylation, and the significance of having multiple H1 subtypes remain to be determined. Histone H1 subtypes are evolutionarily conserved, and are therefore predicted to have different roles [30], even though H1 subtypes can compensate for one another [8]. During the time between activation of the T cells and cell sorting, we found that the relative amounts of the individual subtypes altered, and that the relative content of H1.5 was more than doubled compared with G_0 _T cells (Figure 2; Figure 7A). From the same figures, it is also evident that H1.4 was decreased in activated T cells. However, because of co-migration in HPCE, it is more difficult to state anything about the other subtypes. The subtype composition is believed to be tissue-, developmental- and differentiation-specific [31,32]. Alterations in H1 subtype composition have also been connected to the proliferative activity of mouse cells, in which H1a and H1b (corresponding to human H1.5) were synthesized in large amounts in dividing cells only [33]. Studies of mRNA expression indicated that the levels of H1a, H1b and H1d were reduced in terminally differentiated cells and G_0_-arrested cells [34]. In line with these observations, our results suggest that the H1.5 increase upon T-cell activation is coupled to initiation of proliferative capacity, possibly by priming of chromatin for DNA replication. An intriguing possibility is that a major physiological function of the entire histone H1 protein family and their phosphorylation is to participate in the regulation of local chromatin structure during the cell cycle. If this is true, further exploration of the biological mechanisms behind the extended H1 phosphorylation in G_1 _of malignant cells may provide new targets for cancer therapy in the future.

## Conclusions

Increasing evidence indicates that H1 phosphorylation is important in the priming of chromatin for DNA replication. Our results indicate that an interphase serine phosphorylation pattern becomes largely established during G_1 _or early S phase, and confirm that complementary serine phosphorylation of newly synthesized H1 histones takes place mainly during the S phase of the cell cycle. We also detected a significant increase in the H1.5 content upon activation of T cells, indicating that expression of this subtype may be coupled to proliferative capacity. The T-lymphoblastoid cells showed a more extended H1 phosphorylation in G_1 _compared with normal T cells, which may be a part or a consequence of aberrant cell-cycle control in malignant cells.

## Methods

### Isolation of peripheral blood lymphocytes

Leukocyte-enriched buffy coats from three healthy blood donors were obtained (Blood Bank, Linköping University Hospital, Sweden). Peripheral blood mononuclear cells (PBMCs) were isolated by density-gradient centrifugation (Ficoll-Paque PLUS; GE Heathcare Bio-Sciences, Uppsala, Sweden). Monocytes were removed by plastic adherence during incubation for 1 hour at 37°C and 5% CO_2_, and PBLs were then collected from the supernatants.

### Activation of peripheral blood lymphocytes, cell culture and staining

All media and chemicals were obtained from Gibco (Paisley, Renfrewshire, UK) unless otherwise indicated. After isolation, PBLs were resuspended in RPMI 1640 medium supplemented with 10% v/v fetal bovine serum (FBS), 60 μg/ml penicillin, 100 μg/mL streptomycin, 10 mmol/l HEPES and 2 mmol/l L-glutamine at a concentration of 1 × 10^6 ^cells/mL. The cells were activated by addition of 150 U/ml interleukin (IL)-2 (Proleukin; Chiron Corporation, Emeryville, CA, USA) and1 μg/mL phytohemagglutinin (PHA-M) (Sigma, St Louis, MO, USA).

The cells were counted by trypan blue exclusion, and recultured daily or after 2 to 3 days, depending on cell concentration. Cells were recultured to a cell concentration of 0.5 to 0.6 × 10^6 ^cells/mL in culture medium supplemented with150 U/mL IL-2. PBLs were cultured for 6 to 9 days, depending on the number of cells. The day before sorting, the cells were reconstituted to a concentration of 0.5 to 0.6 × 10^6 ^cells/mL. Before sorting, about 200 to 300 × 10^6 ^PBLs were stained by addition of 10 μg/mL Hoechst 33342 (Molecular Probes, Eugene, OR, USA) into the medium, and incubated in the dark at 37°C and 5% CO_2 _for 30 minutes. The stained cells were subsequently separated by centrifugation at 300 *g *for 10 minutes at 4°C, and the cell pellet resuspended in fresh culture medium to obtain approximately 60 × 10^6 ^cells/mL. The resuspended cells were kept on ice until sorting. This staining procedure was performed in batches two to three times during the cell sorting to minimize the effects of dye exposure, agitation of tubes in the flow cytometer, and prolonged incubation on ice.

### Jurkat cell culture and staining

Jurkat cells (clone E6.1, ECACC, UK) were cultured in RPMI 1640 supplemented with 10% v/v fetal bovine serum (FBS), 60 μg/ml penicillin, 100 μg/mL streptomycin, 2 mmol/l L-glutamine at 37°C and 5% CO_2_. The cells were split three times per week, and kept at a a level of 0.1 to 1 × 10^6 ^cells per mL. The day before sorting, the cells were seeded to 0.25 × 10^6 ^cells per mL so that they were in log growth phase with approximately 0.5 × 10^6^cells per mL upon sorting. The cells were stained with10 μg/mL Hoechst 33342 for 30 minutes in the dark at 37°C and 5% CO_2_. Stained cells were separated by centrifugation at 300 *g *for 10 minutes at 4°C. The cell pellet was resuspended in fresh medium to obtain a cell concentration of approximately 60 × 10^6 ^cells per mL, and kept on ice until sorted. The staining was performed in batches during sorting until sorting was completed.

### T-cell assessments

T cells were assessed for purity, activation and viability by flow cytometry. T-cell purity was determined by measurements of the fraction of CD3+ cells. Cell growth was assessed through cell-cycle analysis of PI-stained cell nuclei; and by cell tracing using CFSE labeling. The amount of CFSE becomes divided between the daughter cells at cell division, which enables determination of the fraction of cycling cells. Cell viability was measured by Annexin V staining. All measurements were done immediately after isolation, at 1 to 3-day intervals post-activation until cell sorting, and at cell sorting.

### CFSE staining and flow-cytometry measurements

After isolation of PBLs, 25 × 10^6^cells were separated by centrifugation at 300 *g*, and washed with PBS supplemented with 1% BSA. Cells were resuspended in 25 ml of 10 μmol/l CFSE (Fluka; Sigma) in PBS plus 1% BSA, and incubated for 10 minutes at 37°C in the dark. After labeling, the cells were washed twice in cold culture medium, and once with cold PBS. After centrifugation, the PBLs were resuspended in culture medium, and activated with IL-2 and PHA-M as described above. For CFSE measurements, 1 × 10^6 ^cells were separated by centrifugation at 300 *g *for 10 minutes and resuspended in 1 ml PBS, after which 15,000 cells were analyzed using a flow cytometer (excitation 488 nm, emission via a BP 530/28 filter) (BD LSR; BD Biosciences). Histograms of CFSE fluorescence, after excluding debris in forward scatter (FSC) and side scatter (SSC), were obtained using CellQuest™ Pro software (BD Biosciences, San Jose, CA, USA). Cells with lower fluorescence than the original fluorescence channel FL1 peak appearing at day 1 were considered as cycling cells.

### CD3 staining and flow-cytometry measurements

The fraction of CD3+ cells in the cell culture was measured using monoclonal anti-human CD3 phycoerythrin (PE) conjugate (Sigma) according to the manufacturer's recommendations. The cells were analyzed for PE fluorescence intensity using a flow cytometer (excitation at 488 nm, emission via a BP 575/26 filter) (BD LSR; BD Biosciences). FSC and SSC were registered, and 15,000 non-gated events were collected. Histograms of PE fluorescence were acquired using CellQuest™ Pro (BD Biosciences).

### Cell-cycle analysis using propidium iodide

Cell-cycle distribution of activated PBLs was determined at various time points using a method developed by Vindelöv [35]. PI fluorescence was measured on a flow cytometer (BD LSR; BD Biosciences) using a BP 575/26 filter. FSC and SSC were also measured after excitation with the argon 488-nm laser; 15,000 non-gated events were collected. Fluorescence histograms of PI were obtained and analyzed with ModFit LT (Verity Software House, Topsham, ME, USA) after gating cell nuclei by FSC and SSC to exclude cell debris of low FSC and SSC.

### Detection of apoptotic peripheral blood lymphocytes

To determine the fraction of apoptotic PBLs in the cell cultures, apoptosis was assessed with Annexin V staining (Annexin V-PE Apoptosis Detection Kit I; BD Biosciences Pharmingen, San Diego, CA, USA) as described previously [27].

### Cell sorting

After Hoechst 33342 staining of activated T cells and Jurkat cells, cells were sorted using a cell sorter (FACSAria Special Order System Cell Sorter; BD Biosciences). During sorting, the samples were kept at 4°C and were continuously agitated. Sorted cells were kept at below 4°C. Hoechst 33342 fluorescence was detected using a 450/50 filter after excitation of a 355 nm UV laser (yttrium-aluminium-garnet (YAG) 20 mW from Coherent, BD Biosciences). FSC and SSC of cells were detected using a 488/10 filter after excitation with a 488 nm laser (Sapphire 100 mW from Coherent, BD BioSciences). Scatter plots of FSC versus SSC and of width (calculated from height of signal) versus area of the Hoechst 33342 signal, and histograms of Hoechst 33342 fluorescence were obtained using FACSDiva software (BD Biosciences). Gating was performed in the FSC/SSC plot to exclude debris and in the Hoechst 33342 area/width plot to exclude cell doublets. The area or height of the Hoechst 33342 fluorescence from the cells present in both these gates was plotted in a DNA histogram, in which sorting gates were created to achieve sorting of the cells into G_1_-, S- and G_2_/M- phase cells with the highest recovery and purity possible (Figure 5A, E). To assess the purity of sorted cells, reanalysis of sorted cell populations was performed at various times during sorting. Continuous sorting using a yield mask was performed, resulting in a sort rate of about 20,000 to 25,000 cells/s and an efficiency of more than 98%. During each experiment, 400 to 600 × 10^6 ^cells were passed through the high-speed sorter, and about 70 to 150 × 10^6 ^G_1 _phase, 12-35 × 10^6 ^S phase and 10 to 30 × 10^6 ^G_2_/M phase cells were sorted out.

### Extraction of H1 histones

H1 histones were extracted from whole cells with perchloric acid as described previously [36].

### Capillary electrophoresis

HPCE was performed on an electrophoresis system (P/ACE 2100; Beckman Instruments) and System Gold software (Beckman Instruments, Palo Alto, CA, USA). This software was also used for determination of peak heights. An untreated capillary was used in all experiments. Protein samples were injected under pressure, and detection was performed by measuring UV absorption at 200 nm. Separation of H1 histones was performed as described previously [37,38]. All runs were performed at constant voltage (12 kV) and at a capillary temperature of 25°C. The peaks in the electropherograms were identified and designated as described previously in detail [27], using the same types of cells.

### Reversed phase high performance liquid chromatography

Separation of whole linker histones was performed on a column (250 mm × 3 mm I.D.; 5 μm particle pore size; 30 nm pore size; end-capped) (Nucleosil 300-5 C_18_; Machery-Nagel, Düren, Germany). The lyophilized proteins were dissolved in water containing 20 mmol/l 2-mercaptoethanol, and whole samples were injected onto the column. The histone H1 sample was separated by chromatography within 30 minutes at a constant flow of 0.35 ml/min with a linear acetonitrile gradient starting (solvent A: solvent B 30:70; solvent A being water containing 0.1% trifluoroacetic acid (TFA), and solvent B being 70% acetonitrile and 0.1% TFA). The concentration of solvent B was increased from 30% to 60% during a period 30 minutes. The peaks in the chromatograms were identified and designated as described previously [23].

## List of abbreviations

BSA: bovine serum albumin; PBS: phosphate-buffered saline.

## Competing interests

The authors declare that they have no competing interests.

## Authors' contributions

AG designed the study, performed cell culturing and flow cytometry, and wrote most of the manuscript; BS performed RP-HPLC and HPCE, and wrote parts of the manuscript; HG performed flow sorting and wrote parts of the manuscript; AL isolated proteins; HL designed RP-HPLC and HPCE analysis, analyzed RP-HPLC and HPCE data, and helped supervise the project; and IR conceived and supervised the project, and wrote the final manuscript. All authors read and approved the final manuscript.
